# Gender and socioeconomic disparities in reasons for not smoking cigarettes among Danish adolescents

**DOI:** 10.1186/s13104-021-05454-6

**Published:** 2021-01-23

**Authors:** Simone Gad Kjeld, Stine Glenstrup, Lotus Sofie Bast

**Affiliations:** National Institute of Public Health, University of Southern Denmark, Studiestraede 6, 1455 Copenhagen K, Denmark

**Keywords:** Smoking, Cigarettes, Adolescents, Reasons, Motivations, Smoking prevention, Tobacco, Non-smoking

## Abstract

**Objective:**

To examine gender and socioeconomic differences in adolescents’ reasons for not smoking cigarettes using self-reported data from Danish 14-year-olds (N = 1,559) collected in 2018. χ^2^-tests were used to assess whether the proportion of students who rated 12 statements as important reasons for not smoking cigarettes differed according to gender and family occupational social class (OSC).

**Results:**

More girls than boys stated that thinking the taste of cigarettes is disgusting, not being allowed to smoke by parents, knowing smoking is dangerous, not being allowed to smoke before the age of 18, not wanting to be addicted to smoking, and that smoking makes you smell bad were important reasons for choosing not to smoke cigarettes. More boys than girls reported exercising a lot and having a partner that does not smoke as important reasons for not smoking cigarettes. More students with a high OSC compared with a low OSC stated exercising a lot and that smoking makes you smell bad were important reasons. In conclusion, reasons for not smoking cigarettes differed substantially across gender and less according to socioeconomic position.

## Introduction

In a smoking preventive perspective, increased knowledge about gender and socioeconomic disparities in reasons for not smoking may help developing initiatives to reduce the youth tobacco incidence—and consequently, narrowing the social inequalities in health. Generally, individuals with a lower socioeconomic position are more likely to start smoking in adolescence and less likely to quit smoking in adulthood [1, 2]; thus, contributing to social disparities in smoking and in health. An increasing priority of smoking preventive measures is to equalize socioeconomic disparities in smoking [3, 4]. However, these efforts might be challenged because health interventions are sometimes easier adopted by individuals from higher socioeconomic backgrounds [5].

Furthermore, research shows that boys are more attracted to smoking cigarettes compared with girls [6, 7]. In a recent study, we found that girls have more positive attitudes towards several elements of smoking prevention initiatives (e.g., rules for smoking at school) [8]. Hence, gender differences exist in both smoking uptake and in the adoption of smoking preventive initiatives.

Research has found adolescents’ main reasons to stay tobacco free were the negative impact of smoking on health, that smoking is disgusting, and that smoking smells bad [9]. Another study found doing sports and getting yellow teeth were important reasons for not smoking—in addition to health concerns and bad smell of cigarettes [10]. Moreover, research indicates non-smoking adolescents have more knowledge about health-related issues due to smoking compared with adolescents who smoke cigarettes [11]. Studies focusing on socioeconomic and gender differences in reasons for not smoking are, however, very limited. One qualitative study indicated non-smoking boys and girls did not differ markedly in their attitudes towards smoking and across gender, concerns about health and a positive self-image were important reasons for not smoking [12].

Insights into gender and socioeconomic disparities in reasons for not smoking is essential—both for shaping smoking preventive interventions to ensure they reach all adolescents as well as for understanding which parameters may equalize gender and socioeconomic disparities in smoking—and consequently in health. Therefore, the aim of this study was to investigate whether there are gender and socioeconomic disparities in which reasons adolescents’ rate as important for not smoking cigarettes.

## Main text

### Methods

#### Study design

In this study, we used data from the X:IT II intervention, a multi-component intervention aiming at preventing smoking uptake among adolescents in the school [3]. The Danish Cancer Society developed the intervention and its effectiveness was evaluated by the Centre for Intervention Research, University of Southern Denmark from September 2017 until June 2020. From 31 municipalities across Denmark, 300 schools were randomly selected and invited to participate in the study of which 57 schools were eligible for enrollment. After enrollment, 11 schools withdrew from the study due to reasons mainly centering around lack of time and other priorities which left 46 schools that were included at baseline. The enrolled students were followed from the beginning of 7th grade (baseline measurement) until the end of 9th grade (third follow-up measurement), thus, including students from 13 to 15 years of age [3].

Students’ responses to the first follow-up questionnaire were used after initiation of the X:IT II intervention (collected at the end of 7th grade). In total, 44 schools were included in this follow-up measurement as two schools withdrew their participation after the baseline data collection. All students (around 14 years of age) at the schools were encouraged to participate in the data collection which consisted of an internet-based self-reported questionnaire that the students could fill in during school hours with instructions by their teachers. The questionnaire included topics related to students’ as well as their families’ and friends’ tobacco patterns, attitudes and norms towards smoking, sociodemographic items, items about their well-being, and items about the X:IT II intervention and its components.

Of all eligible students (n = 2228), 74.8% (n = 1666) answered the questionnaire. Due to a small number of students identifying as neither a boy nor a girl in the data material (1.6%, n = 27), the group was excluded for further analyses in this study. We also excluded students who currently smokes cigarettes (4.8%, n = 80) as the focus of this study was adolescents’ reasons for *not* smoking cigarettes. Thus, the total study sample here was N = 1,559 (70.0% of eligible students).

#### Measures

*Gender* was assessed by asking the question “are you a boy or a girl?” divided into 1 = boy, 2 = girl, or 3 = students who felt they did not fit into neither of the two first categories. Students in category 3 were excluded for further analyses.

*Family occupational social class (OSC)* was determined by two questions concerning the occupations of students’ father and mother. OSC was coded in accordance with the Danish Occupational Social Class Measurement [13]. Information about parental occupation was categorized from I = high to V = low social class as well as VI = parents receiving social benefits. The parent ranking highest determined the OSC of parents. In this study, OSC was recoded into three categories: 1 = high (I–II), 2 = medium (III–IV), 3 = low (V–VI), and 4 = non-classifiable. Students with a non-classifiable OSC (14.3%) were excluded in the main analyses of the associations between reasons for not smoking and OSC.

*Reasons for not smoking* included twelve statements addressing students who currently do not smoke cigarettes. Students had to rank each statement as either 1 = a very important reason for not smoking, 2 = an important reason for not smoking, 3 = not an important reason for not smoking, and 4 = not an important reason for smoking at all. The variables were dichotomized into 1 = important reason (1 + 2) and 2 = not important reason (3 + 4). The statements are outlined in Table 2.

#### Analyses

The 9.4 version of SAS was used for the statistical analyses. We compared the distributions of variables concerning reasons for not smoking in relation to students’ gender and OSC assessed by χ^2^-tests. A *p*-value of < 0.05 was considered statistically significant.

### Results

As presented in Table 1, almost half of the respondents were girls (51.3%). Concerning family occupational social class (OSC), 38.0% of the students had a high OSC, while 39.0% had a medium, 8.6% had a low, and 14.3% had a non-classifiable OSC (see also Table 1 for all student characteristics).

**Table 1:** Characteristics of students (N = 1,559)

Student characteristics	%	n
Girls	51.3	746
Family occupational social class (OSC)		
High	38.0	542
Medium	39.0	556
Low	8.6	123
Non-classifiable	14.3	204
Ethnicity		
Danish origin	92.6	1102
Descendants of immigrants	4.0	47
Immigrants	2.0	24
Non-classifiable	1.4	17
Family structure		
Lives with two biological parents	68.6	1059
Single-parent family	8.0	124
Reconstituted family	16.3	252
Other (e.g., foster care or institution)	7.0	108
Ever smoked cigarettes	9.9	162
Parental smoking	31.9	493
Best friend’s smoking	10.4	160
Other friends’ smoking	28.5	437
Binge drinking within the last month	17.7	274
Ever tried electronic cigarettes	9.4	146
Ever tried snus	1.4	21
Ever tried water pipe	6.5	100

In Table 2, the proportion of students who reported each of the 12 statements as important reasons for not smoking were compared across gender and OSC. Overall, knowing smoking is dangerous, not wanting to be addicted to smoking, smoking makes you smell bad, and smoking cigarettes is not interesting were most frequently reported as important reasons for not smoking across OSC and gender.

**Table 2:** Proportion of boys and girls as well as students with a high, medium, and low OSC who have reported each of the twelve statements as important reasons for not smoking

	Boys (%)	Girls (%)	*p*-value	High OSC (%)	Medium OSC (%)	Low OSC (%)	*p*-value
None of my friends smoke	39.2	34.5	0.081	36.5	36.1	36.4	0.994
I think the taste of cigarettes is disgusting	61.8	70.0	*0.002*	68.9	65.0	60.2	0.159
I am not allowed to smoke by my parents	71.0	80.1	* < 0.001*	79.2	74.4	72.1	0.111
I think smoking is too expensive	79.2	77.0	0.349	78.7	75.5	78.2	0.475
I know smoking is dangerous	89.8	96.3	* < 0.001*	95.4	91.4	92.7	*0.043*
Smoking is not interesting	82.1	84.1	0.341	82.2	84.0	81.8	0.692
You are not allowed to smoke before the age of 18	56.8	63.9	*0.009*	60.2	58.1	67.9	0.164
I exercise a lot	65.4	59.6	*0.033*	67.3	58.9	57.4	*0.013*
I like making my own decisions	64.3	66.3	0.447	65.0	66.8	58.7	0.275
My partner does not smoke	40.0	31.8	*0.003*	35.4	35.4	32.1	0.796
I do not want to be addicted to smoking	83.7	90.6	* < 0.001*	89.8	84.5	86.2	*0.044*
Smoking makes you smell bad	81.0	87.6	*0.001*	86.5	83.4	75.9	*0.022*

*p*-values are marked with italics when significant at the 5% level

Results show that more girls than boys reported thinking the taste of cigarettes is disgusting (girls: 70.0%, boys: 61.8%, *p* = 0.002), not being allowed to smoke by their parents (girls: 80.1%, boys: 71.0%, *p* < 0.001), knowing smoking is dangerous (girls: 96.3%, boys: 89.8%, *p* < 0.001), not being allowed to smoke before the age of 18 (girls: 63.9%, boys: 56.8%, *p* = 0.009), not wanting to be addicted to smoking (girls: 90.6%, boys: 83.7%, *p* < 0.001), and that smoking makes you smell bad (girls: 87.6%, boys: 61.8%, *p* = 0.002) were important reasons for not smoking. On the other hand, more boys than girls reported exercising a lot (girls: 70.0%, boys: 61.8%, *p* = 0.002) and having a partner that does not smoke (girls: 70.0%, boys: 61.8%, *p* = 0.002) as important reasons for not smoking cigarettes.

We found that more students with a high OSC compared with a low OSC reported exercising a lot (high OSC: 67.3%, low OSC: 57.4%, *p* = 0.013) and that smoking makes you smell bad (high OSC: 86.5%, low OSC: 75.9%, *p* = 0.022) were important reasons for not smoking.

## Discussion

This study is among the few which have studied gender and socioeconomic differences in young people’s reasons for not smoking cigarettes. We found that students’ reasons for not smoking differed quite substantially across gender, while the differences were less clear across socioeconomic positions.

Across gender and socioeconomic positions, we found that students frequently reported knowing smoking is dangerous, not wanting to be addicted to smoking, thinking smoking makes you smell bad, and smoking cigarettes is not interesting as important reasons for not smoking cigarettes. These findings are consistent with existing literature which have indicated health concerns and the bad smell of cigarettes as main reasons for not smoking cigarettes among the youth [9]. Moreover, research indicated that thinking cigarettes are disgusting was an additional important reason for not smoking [9, 10], while in this study, this reason was mostly reported as important by girls.

The only identified study addressing gender differences in reasons for not smoking indicated that concerns about health were equally important reasons for not smoking across gender [12]. In contrast, our study found girls more often than boys reported knowing smoking is dangerous and not wanting to be addicted to smoking as important reasons for not smoking. This might be due to boys not having the same risk assessment about the danger of smoking [14] or other factors may be more important for boys’ motivations to not smoke. For example, in this study, boys reported exercising a lot was an important reason for not smoking. In this connection, a study found that boys smoking cigarettes were especially concerned about the effect of smoking on their fitness and sport, while girls were more concerned about the smell of cigarettes [15].

More girls than boys reported in this study that regulations about smoking (i.e., not being allowed to smoke by parents and not being allowed to smoke before the age of 18) were important reasons for not smoking. These findings are in line with existing research indicating that girls and young females have more positive attitudes towards smoking regulations—both at the societal and school level as well as at home [8, 16].

We did not find substantial differences across socioeconomic positions in reasons for not smoking, although there was a tendency to more students with a high OSC reporting exercising a lot as an important reason for not smoking. This relates to existing literature indicating that adolescents with higher socioeconomic positions are more physically active [17]; thus, being physically active may impact students’ decisions about smoking. Thinking smoking makes you smell bad was also an important reason for not smoking among adolescents with a high OSC relative to adolescents with a medium or low OSC. More research about socioeconomic disparities in reasons for not smoking is needed to qualify these findings.

## Limitations

An important strength of this study is the school-based evaluation design—as most children and adolescents attends public or private schools in Denmark, the possibility of reaching all students regardless of gender and socioeconomic positions is high. Nonetheless, this study may also have some limitations.

One limitation of this study may be that a specific group of adolescents are more likely to answer the questionnaire than others; thus, this study may have some degree of self-selection bias. In a recent study, however, we found that schools participating in the X:IT II study were not markedly different compared with all schools in Denmark on several parameters, including organizational resources, student enrollment, ethnic composition, average grades, and academic well-being. The biggest difference seemed to be that more public schools than private schools participated in the X:IT II study [18].

In this study, we used self-reported data from students as we believe that this information provides the most in-depth insights into adolescents’ everyday lives. Validation studies have found that adolescents’ self-reporting of smoking is consistent with biochemical measures of tobacco consumption [19–21]. However, these studies also indicated that a group of adolescents who reported not to smoke may in fact be smoking.

Another limitation may be that students were only able to assess whether they believed the 12 statements were important reasons for not smoking. Because students were not able to write if they considered other reasons for not smoking important, we may have missed some important information.

Approximately 14% of the students were categorized with a non-classifiable OSC and in this study, we chose to exclude this group from the analyses due to the heterogeneity of the group. The proportion of students that could not be classified within the low, medium, or high OSC groups may bias our findings and therefore, we recommend that future research will further investigate how reasons for not smoking potentially differ across socioeconomic positions.

## Data Availability

Data that support the findings of this study are available from University of Southern Denmark (SDU). Restrictions apply to the availability of data which were used under license for the current study and are thus, not publicly available. However, data are available from the corresponding author upon reasonable request and with permission of SDU.

## Abbreviations

OSC: Family occupational social class
